# Melatonin ameliorates tau-related pathology via the miR-504-3p and CDK5 axis in Alzheimer’s disease

**DOI:** 10.1186/s40035-022-00302-4

**Published:** 2022-05-09

**Authors:** Dongmei Chen, Guihua Lan, Ruomeng Li, Yingxue Mei, Xindong Shui, Xi Gu, Long Wang, Tao Zhang, Chen-Ling Gan, Yongfang Xia, Li Hu, Yuan Tian, Mi Zhang, Tae Ho Lee

**Affiliations:** 1 Fujian Key Laboratory of Translational Research in Cancer and Neurodegenerative Diseases, Institute for Translational Medicine, School of Basic Medical Sciences, Fujian Medical University, Fuzhou, 350122 Fujian China; 2 Department of Pathology, Longyan First Hospital Affiliated to Fujian Medical University, Longyan, 364000 Fujian China

**Keywords:** Alzheimer’s disease, Melatonin, miR-504-3p, p39, Tau

## Abstract

**Background:**

Intracellular accumulation of the microtubule-associated protein tau and its hyperphosphorylated forms is a key neuropathological feature of Alzheimer’s disease (AD). Melatonin has been shown to prevent tau hyperphosphorylation in cellular and animal models. However, the molecular mechanisms by which melatonin attenuates tau hyperphosphorylation and tau-related pathologies are not fully understood.

**Methods:**

Immunofluorescence, immunoblotting analysis and thioflavin-S staining were employed to examine the effects of early and late treatment of melatonin on tau-related pathology in hTau mice, in which nonmutated human tau is overexpressed on a mouse tau knockout background. High-throughput microRNA (miRNA) sequencing, quantitative RT-PCR, luciferase reporter assay and immunoblotting analysis were performed to determine the molecular mechanism.

**Results:**

We found that both early and late treatment of melatonin efficiently decreased the phosphorylation of soluble and insoluble tau at sites related to AD. Moreover, melatonin significantly reduced the number of neurofibrillary tangles (NFTs) and attenuated neuronal loss in the cortex and hippocampus. Furthermore, using miRNA microarray analysis, we found that miR-504-3p expression was upregulated by melatonin in the hTau mice. The administration of miR-504-3p mimics dramatically decreased tau phosphorylation by targeting p39, an activator of the well-known tau kinase cyclin-dependent kinase 5 (CDK5). Compared with miR-504-3p mimics alone, co-treatment with miR-504-3p mimics and p39 failed to reduce tau hyperphosphorylation.

**Conclusions:**

Our results suggest for the first time that melatonin alleviates tau-related pathologies through upregulation of miR-504-3p expression by targeting the p39/CDK5 axis and provide novel insights into AD treatment strategies.

**Supplementary Information:**

The online version contains supplementary material available at 10.1186/s40035-022-00302-4.

## Background

Alzheimer's disease (AD) is one of the most common neurodegenerative diseases characterized by progressive mental decline and is a leading cause of dementia in the elderly [1, 2]. Intracellular neurofibrillary tangles (NFTs) composed of heavily phosphorylated forms of tau protein and extracellular senile plaques consisting of beta-amyloid (Aβ) peptides in the brain are the major histopathological features of AD [3]. NFTs are closely related to extensive neuronal loss in AD [4]. As a microtubule-associated protein, tau stimulates the assembly of tubulin into microtubules, thereby stabilizing microtubules in the brain [5, 6]. Hyperphosphorylated tau forms paired helical filaments with aberrant conformations, which affect the binding of tau to microtubules and inhibit tubulin assembly, leading to microtubule dysfunction and protein stability disruption, eventually forming NFTs during neurodegeneration in AD [5–8]. Therefore, a therapeutic strategy to inhibit tau hyperphosphorylation in AD is urgently needed.

Currently, more than 40 phosphorylation sites of tau have been discovered in the brains of AD patients [9, 10]. Tau phosphorylation/dephosphorylation is controlled by several protein kinases and phosphatases [11, 12]. Thus, dysregulation of protein kinases and phosphatases leads to tau-related pathology in AD. Multiple protein kinases have been implicated in the phosphorylation of tau in AD [11, 12]. For example, cyclin-dependent kinase 5 (CDK5) has been demonstrated to phosphorylate tau at Ser202, Ser235, Ser396 and Ser404, as well as Thr205 and Thr231 [8, 13]. Takahashi et al. showed that CDK5/p39 is responsible for tau phosphorylation at Ser202 and Thr205 [14]. The binding of CDK5 to its specific binding partners p35 and p39 or their cleavage products (p25 and p29, respectively) results in CDK5 activation [15]. CDK5 promotes hyperphosphorylation of tau and the formation of NFTs, while inhibition of CDK5 attenuates memory deficits by reducing tau phosphorylation [16]. Microtubule affinity-regulating kinase (MARK) 1–4 are tau kinases that phosphorylate tau at Ser262 and Ser356 [17, 18]. CDK5 enhances the phosphorylation of tau at Ser262 and the accumulation of tau by activating MARK4, consequently promoting tau-induced neurodegeneration [18]. Although CDK5 has an important effect on tau hyperphosphorylation in AD, the upstream factors involved and the mechanisms underlying the CDK5-mediated tau hyperphosphorylation are not fully known.

Melatonin, an indole neuroendocrine hormone mainly synthesized in the pineal gland, can easily cross the blood–brain barrier [19, 20]. It has been reported that melatonin plays neuroprotective roles in neurodegenerative diseases, including AD [20–26]. Moreover, melatonin levels are decreased in the cerebrospinal fluid (CSF), serum and brains of AD patients compared with age-matched healthy people [27–30]. Melatonin has been shown to efficiently suppress tau hyperphosphorylation in neuronal cell lines and animal models induced by Aβ peptides, tau mutants, kainic acid, okadaic acid, calyculin A and wortmannin [21, 30–36]. However, whether melatonin affects tau hyperphosphorylation and tau-related pathologies in mice in which the mouse tau gene is replaced by overexpression of wild-type (WT) human tau has not been well studied. Moreover, the mechanisms underlying the melatonin-mediated attenuation of tau hyperphosphorylation in AD have not been fully elucidated.

Previous studies have shown that the expression of microRNAs (miRNAs) is altered in neurodegenerative diseases, including AD [37–39]. MiRNAs have been shown to regulate the expression or activity of protein kinases and phosphatases, thereby affecting tau phosphorylation [39–44]. For example, it has been observed that the overexpression of miR-26b causes the transport of CDK5 from the nucleus to the cytoplasm and the activation of CDK5 via targeting of the retinoblastoma protein, which induces tau hyperphosphorylation [39]. These results suggest that neuronal miRNA dysfunction might be an important factor contributing to AD pathology. Several studies have shown that the expression of a large number of miRNAs is modulated by melatonin and that many miRNAs contribute to circadian rhythms and exert anticancer and antioxidative effects [45–49]. Recent research found that melatonin increases the transcription levels of several miRNAs, including miR-171b, which targets α-glucan water dikinase, thereby ameliorating the carbon starvation-induced leaf senescence in tomatoes [50]. Melatonin has been shown to protect against congenital spinal deformities by inhibiting miR-363 expression and upregulating Notch signaling [46]. Moreover, it has been reported that melatonin exerts a neuroprotective effect on Aβ-induced neuronal damage in primary neurons by restoring miR-132 expression and downregulating PTEN and FOXO3a levels [51]. Melatonin ameliorates Aβ-induced dendritic abnormalities and learning and memory deficits in AD via downregulation of miR-125b through melatonin receptor 2 [52]. Melatonin has also been found to attenuate the scopolamine-induced synaptic and memory disorders in AD by suppressing the expression of miR-124 [53]. However, whether melatonin protects against tau hyperphosphorylation in AD through the modulation of miRNA expression remains largely unknown.

In this study, we set out to study the effect of melatonin on tau-related pathologies including tau hyperphosphorylation, NFT formation, and neuronal loss using transgenic mice expressing WT human tau. We compared miRNA expression profiles between saline- and melatonin-treated transgenic mice expressing WT human tau by high-throughput sequencing, and examined the potential mechanistic link between a melatonin-regulated miRNA and AD.

## Materials and methods

### Reagents

*N*-lauroylsarcosine sodium salt was purchased from Macklin (Shanghai, China) and was used to isolate sarkosyl-insoluble fractions. Thioflavin-S was obtained from MilliporeSigma (St. Louis, MO) and was used to determine the number of NFTs. Melatonin was purchased from Aladdin (Shanghai, China), and protein lysis buffer was purchased from Beyotime (Shanghai, China). Protease inhibitor and phosphatase inhibitor cocktails were purchased from TransGen Biotech (Beijing, China).

### Animals and drug treatment

**Fig. 1:**
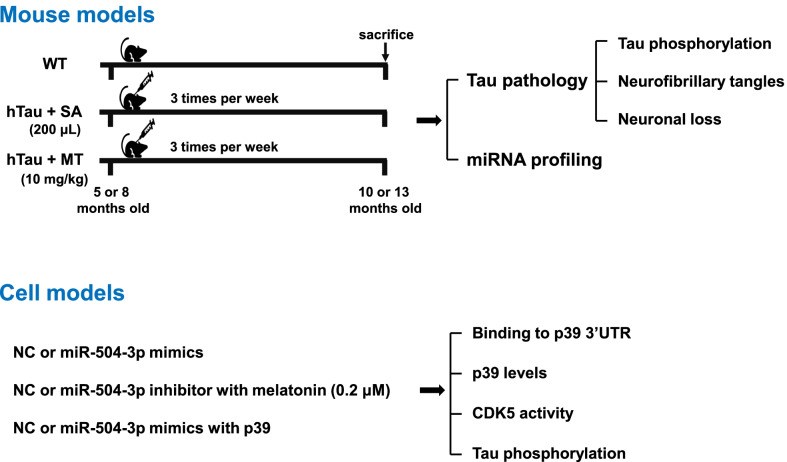
The experimental design. Melatonin or saline was subcutaneously administered to 5- and 8-month-old male hTau mice 3 times per week for 5 months. The mice were subjected to tau pathology examination and miRNA profiling. N2a cells were treated with NC, miR-504-3p mimics, or miR-504-3p inhibitor in the absence or presence of melatonin or p39 and then subjected to functional and mechanistic studies

WT and hTau mice were obtained from the Shanghai Laboratory Animal Center (Shanghai, China) and Jackson Laboratory (Bar Harbor, ME), respectively. All mice were on the C57BL/6 background, and were maintained on a 12-h light/dark cycle with continuously available food and water. All animal experiments were approved by the Experimental Animal Ethics Committee of Fujian Medical University (FJMUIACUC 2021-J-0094). Melatonin (10 mg/kg) or saline (200 μl) was subcutaneously administered to 5- and 8-month-old male hTau mice 3 times per week for 5 months (Fig. 1). Untreated male WT mice were used as controls.

### Brain samples

The mice were anesthetized by intraperitoneal injection of 2% pentobarbital sodium (45 mg/kg). Whole-brain tissues (left hemisphere) and hippocampal and cortical tissues (right hemisphere) were harvested from WT mice, as well as saline-treated and melatonin-treated hTau mice at 10 and 13 months of age. After extraction, the hippocampal and cortical tissues, which were used for immunoblotting analysis or miRNA sequencing, were stored at − 80 °C, while whole-brain tissues, which were used for immunohistochemical analysis, were embedded in paraffin and stored at 4 °C.

### Extraction of sarkosyl-soluble and sarkosyl-insoluble tau

The sarkosyl-soluble and sarkosyl-insoluble fractions of mouse brain tissues were isolated as reported previously with minor modifications [54]. The brain tissues were homogenized in lysis buffer (500 μl/30 mg) containing protease inhibitor and phosphatase inhibitor cocktails (1 mM) on ice for 30 min. The supernatant was collected after centrifugation at 12,000 g at 4 °C for 20 min, mixed with 1% (*w*/*v*) *N*-lauroylsarcosine and incubated at room temperature for 1 h. The mixture was then centrifuged at 100,000 g at 4 °C for 1 h. The supernatant was collected as the sarkosyl-soluble fraction, while the pellet was suspended in 50 mM Tris–HCl (pH 7.4) at 4 °C for 2 h and used as the sarkosyl-insoluble fraction.

### Cell culture

The mouse neuroblastoma cell line N2a and human embryonic kidney cell line 293T were purchased from the Stem Cell Bank/Stem Cell Core Facility (Shanghai, China). The cells were maintained in high-glucose Dulbecco’s modified Eagle’s medium containing 10% fetal bovine serum, 100 U/ml penicillin and 100 µg/ml streptomycin (Thermo Fisher Scientific, Waltham, MA). The cells were cultured at 37 °C and 5% CO_2_.

### Immunoblotting analysis

Proteins were isolated by lysing cells in lysis buffer containing 50 mM Tris–HCl (pH 7.4), 1% Triton X-100, 50 mM NaCl, 1 mM DTT, and 1 mM EGTA in the presence of protease and phosphatase inhibitor cocktails (1 mM). The protein concentration was measured using a BCA protein assay kit (Beyotime). Protein samples (5–15 μg) were transferred onto polyvinylidene fluoride membranes (MilliporeSigma) after being separated by SDS-PAGE. The membranes were blocked with 5% BSA-TBST or 5% milk-TBST at room temperature for 1 h and then incubated with various primary antibodies at 4 °C overnight, followed by HRP-conjugated secondary antibodies at room temperature for 1 h. Finally, the proteins were detected using ECL chemiluminescent HRP substrate (MilliporeSigma) using a Bio-Rad ChemiDoc imaging system. The protein levels were quantified by the intensity of the grayscale images using the ImageJ software (version 1.50i, NIH, USA), and were normalized to β-actin. Two types of p39 antibodies were used to measure endogenous p39 levels (Abcam, Waltham, MA) as well as both endogenous and overexpressed p39 levels (Cell Signaling Technology, Danvers, MA). The primary antibodies used in this study are listed in Additional file 1: Table S1.

### CDK5 activity assay

The CDK5 kinase assay was performed as described with modifications [55]. Cell lysates were prepared with lysis buffer and immunoprecipitated with a CDK5 antibody. The immunoprecipitates were suspended in CDK5 kinase buffer (50 mM HEPES pH 7.5, 0.5 mM DTT, 5% glycerol) in the presence of 90 mM ATP, 0.1 mM CDK5 substrate, glutathione-S-transferase (GST)-C-terminal amyloid precursor protein (APP) (GST- KKKQYTSIHHGVVEVDAAVTPEERHLSKMQQNGYENPTYKFFEQMQN) at room temperature for 30 min. The protein samples were then subjected to immunoblotting analysis with an anti-pThr668 APP antibody.

### Immunofluorescence analysis

Tissue sections were deparaffinized with xylene, rehydrated in a descending ethanol series and washed with ddH_2_O. After antigen retrieval, the sections were blocked with PBS containing 10% FBS and 0.05% Tween-20 at room temperature for 40 min. After incubation with primary antibodies at 4 °C overnight, the slides were incubated with Alexa Fluor 488- or 546-conjugated secondary antibodies (Thermo Fisher Scientific) at room temperature for 1.5 h. Finally, the sections were stained with Hoechst 33342, covered with antifade mounting medium, and analyzed by microscopy. Tau phosphorylation was quantified with the ImageJ software.

### Thioflavin-S staining

Paraffin-embedded tissue slices were washed with PBS for 15 min and stained with 0.3% thioflavin-S in 50% ethanol at room temperature for 8 min. After rinsing with 50% ethanol 3 times, the slices were incubated in PBS for 5 min. The number of NFTs was determined by counting the number of NFTs in a single brain image from each mouse using the ImageJ software.

### Quantitative RT-PCR

Total RNA was isolated using NucleoZOL (Macherey–Nagel, Duren, Germany) following the manufacturer’s instructions. cDNA was synthesized from miRNA and mRNA using a One-Step miRNA cDNA Synthesis Kit (HaiGene, Haerbin, China) and a Transcriptor First Strand cDNA Synthesis Kit (Roche, Indianapolis, IN), respectively. Real-time qRT-PCR was performed using FastStart Universal SYBR Green Master Mix (Roche) on the QuantStudio Real-Time PCR System (Thermo Fisher Scientific) according to the manufacturer’s instructions. To amplify mmu-miR-504-3p, the following primers were used: forward, 5′-GGGAGAGCAGGGCAG-3′; reverse, 5′-GTCCAGTTTTTTTTTTTTTTTGAAACC-3′. For p39 amplification, the following primers were used: forward, 5′-AACCTGGTGTTCGTGTACCTGCT-3′; reverse, 5′-AGATCTCGTTGCCCATGTAGGAGT-3′. For amplification of U6, the following primers were used: forward, 5′-CTCGCTTCGGCAGCACA-3′; reverse, 5′-AACGCTTCACGAATTTGCGT-3′. For amplification of 18S rRNA, the following primers were used: forward, 5′-TGTCTCAAAGATTAAGCCATGCA-3′; reverse, 5′-GCGACCAAAGGAACCATAACTG-3’. Each sample was amplified in duplicate. Data were analyzed by the comparative Ct (ΔΔCt) method by normalizing expression to the U6 level for miRNA and to the 18S rRNA level for mRNA.

### Luciferase reporter assay

To assess the interaction between miR-504-3p and the p39 3′UTR, the WT p39 3′UTR or a mutant p39 3′UTR containing the predicted miR-504-3p-binding site was cloned into the pmirGLO luciferase vector (Promega, Fitchburg, WI). Plasmids containing the WT 3′UTR or mutant 3′UTR were cotransfected with a negative control (NC) or miR-504-3p mimics (GenePharma, Shanghai, China) into 293T cells using TurboFect transfection reagent (Thermo Fisher Scientific). Luciferase activity was measured using the Dual-Luciferase Reporter Assay System (Promega) according to the manufacturer’s protocol.

### MiRNA sequencing

Total RNA was isolated from the hippocampi of saline- and melatonin-treated hTau mice at 10 months of age, and the miRNAs were sequenced (RIBOBIO, Guangzhou, China). The RNA quantity and purity were examined using a NanoDrop K5500 spectrophotometer, while the RNA integrity was assessed by the Agilent 2200 RNA assay and gel electrophoresis. An A260/A280 ≥ 1.5 and A260/A230 ≥ 1.0 indicated acceptable RNA purity, while an RNA integrity number (RIN) ≥ 7 and 28 S/18 S ≥ 1.0 indicated acceptable RNA integrity. The miRNA expression profiles are listed in Additional file 2.

### Statistical analysis

Statistical analysis was carried out using the GraphPad Prism 7.0 software. The experimental data are presented as the means ± standard errors of three independent experiments. A two-tailed unpaired *t*-test was applied to analyze the statistical significance of differences between two groups, while one-way analysis of variance (ANOVA) followed by Tukey's multiple comparisons test was applied for comparison of multiple groups. *P* < 0.05 was considered as statistically significant.

## Results

### Melatonin reduces tau hyperphosphorylation in hTau mice

**Fig. 2:**
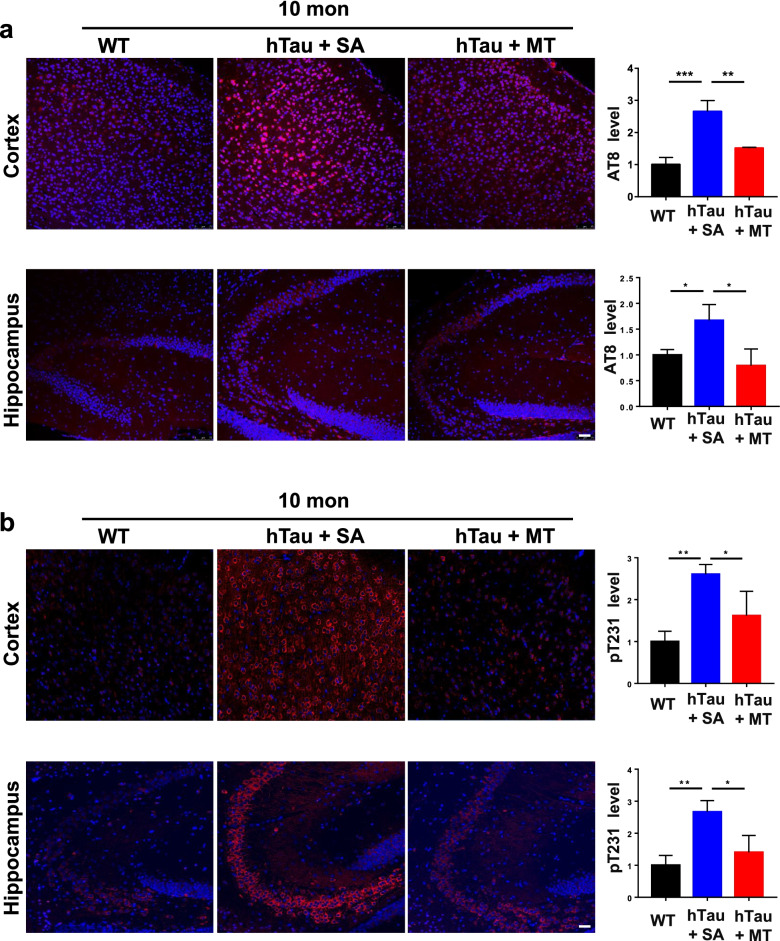
Melatonin decreases tau phosphorylation in 10-month-old hTau mice. Immunofluorescence using an AT8 (epitopes for pSer202/pThr205 tau) (**a**) or an anti-pThr231 tau (**b**) antibody was performed on paraffin-embedded cortical and hippocampal sections from WT (*n* = 8), saline-treated hTau (hTau + SA) (*n* = 5) and melatonin-treated hTau (hTau + MT) (*n* = 5) mice. Scale bars, 50 μm. Data are presented as the means ± standard errors (*P < 0.05, **P < 0.01, ***P < 0.001)

**Fig. 3:**
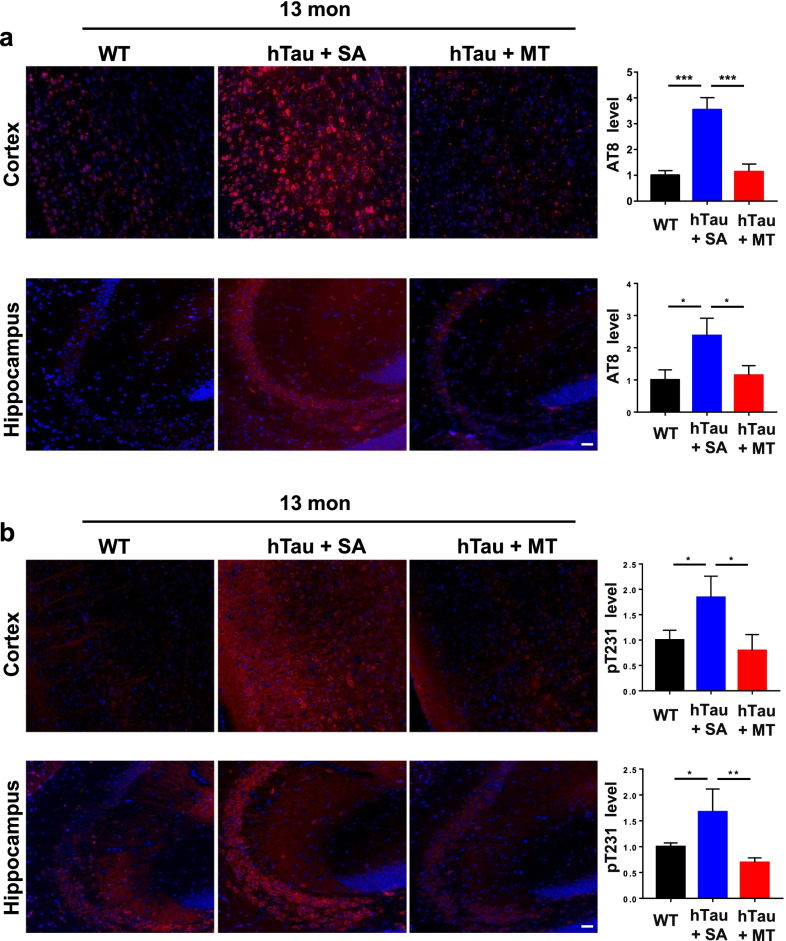
Melatonin decreases tau phosphorylation in 13-month-old hTau mice. Immunofluorescence using an AT8 (epitopes for pS202/T205-Tau) (**a**) or anti-pT231 Tau (**b**) antibody was performed on paraffin-embedded cortical and hippocampal sections from WT (*n* = 8), saline-treated hTau (hTau + SA) (*n* = 5) and melatonin-treated hTau (hTau + MT) (*n* = 5) mice. Scale bars, 50 μm. Data are presented as the means ± standard errors (*P < 0.05, **P < 0.01, ***P < 0.001)

**Fig. 4:**
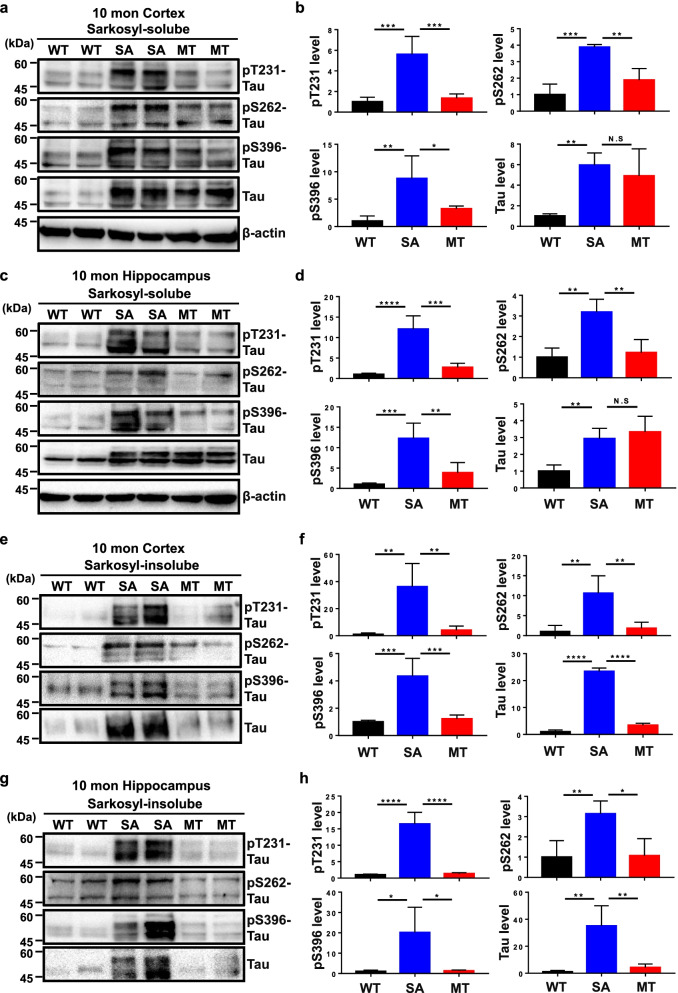
Melatonin reduces tau hyperphosphorylation and the formation of sarkosyl-insoluble tau aggregates in 10-month-old hTau mice. Cortical and hippocampal tissues from WT (*n* = 8), hTau + SA (*n* = 5) and hTau + MT mice (*n* = 5) were separated into sarkosyl-soluble (**a–d**) and sarkosyl-insoluble fractions (**e–h**). The protein samples were subjected to immunoblotting analysis with anti-pThr231 tau, anti-pSer262 tau, anti-pSer396 tau, anti-tau or anti-β-actin antibody. Sarkosyl-soluble β-actin was used as an internal control and for normalization in both the sarkosyl-soluble and -insoluble fractions. Data are presented as the means ± standard errors (*P < 0.05, **P < 0.01, ***P < 0.001, ****P < 0.0001; N.S., no significance)

**Fig. 5:**
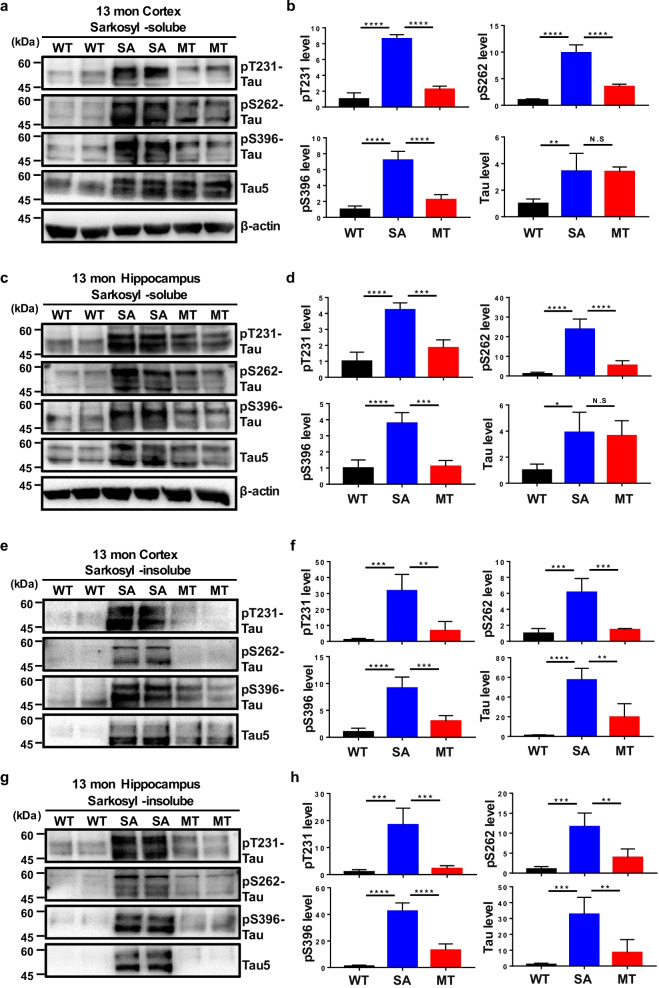
Melatonin reduces tau phosphorylation and the formation of sarkosyl-insoluble tau aggregates in 13-month-old hTau mice. Cortical and hippocampal tissues from WT (*n* = 8), hTau + SA (*n* = 5) and hTau + MT mice (*n* = 5) were separated into sarkosyl-soluble (**a–d**) and sarkosyl-insoluble fractions (**e–h**). The protein samples were subjected to immunoblotting analysis with anti-pThr231 Tau, anti-pSer262 Tau, anti-pSer396 Tau, anti-tau or anti-β-actin antibodies. Sarkosyl-soluble β-actin was used as an internal control and for normalization in both the sarkosyl-soluble and -insoluble fractions. Data are presented as the means ± standard errors (*P < 0.05, **P < 0.01, ***P < 0.001, ****P < 0.0001; N.S., no significance)

Previous studies by our group and others have found that melatonin decreases tau phosphorylation in vitro and in vivo [30, 32, 34, 56]. However, the effect of melatonin on tau phosphorylation in animal models expressing human tau without mutations that induce human AD-like and tau-related pathologies has not been investigated. We aimed to clarify whether melatonin attenuates tau hyperphosphorylation in hTau mice, which were generated by crossing mice expressing all WT human tau isoform transgenes with mice with tau ablation [57]. Given that the hTau mice exhibit significant tau hyperphosphorylation and tau-related pathologies in an age-dependent manner [57, 58], melatonin was administered at two time points. Since hyperphosphorylated tau accumulation begins in hTau mice from 6 months of age, we administered saline or melatonin to 5- and 8-month-old mice for 5 months to study the effects of early and late treatment with melatonin. In contrast to saline, melatonin decreased the phosphorylation of tau in the cortices and hippocampi of hTau mice at both 10 and 13 months of age, as measured by immunohistochemistry using an AT8 antibody (epitopes for pSer202/pThr205) and a pThr231-specific antibody (Figs. 2 and 3). The aberrantly phosphorylated tau protein aggregates and forms fibrils with irregular conformations in diseases involving tau pathology, which indicates that the change of tau structure from the normal soluble form to the filamentous insoluble form is pathological. Thus, sarkosyl-soluble and -insoluble fractions, which contained phosphorylated tau and abnormal fibrils, respectively, were isolated. The levels of phosphorylated tau but not the total tau level was significantly decreased in the sarkosyl-soluble fraction of cortical and hippocampal tissues from melatonin-treated hTau mice compared with those from saline-treated hTau and WT mice, at both 10 and 13 months of age (Figs. 4a–d, 5a–d), whereas the levels of both the total and the phosphorylated tau in the sarkosyl-insoluble fraction were dramatically decreased in the melatonin group compared to the saline group, at both 10 and 13 months of age, as measured by immunoblotting using pThr231-, pSer262- and pSer396-specific antibodies (Figs. 4e–h, 5e–h). Moreover, the phosphorylated tau in the sarkosyl-insoluble fraction was reduced to a much greater extent than that in the sarkosyl-soluble fraction. These results suggest that both early and late treatment with melatonin can reduce tau phosphorylation, the total tau level, and abnormal tau aggregation, indicating that melatonin may have both preventive and curative effects in the brain tissues of hTau mice.

### Melatonin attenuates NFT formation and alleviates neuronal loss in hTau mice

**Fig. 6:**
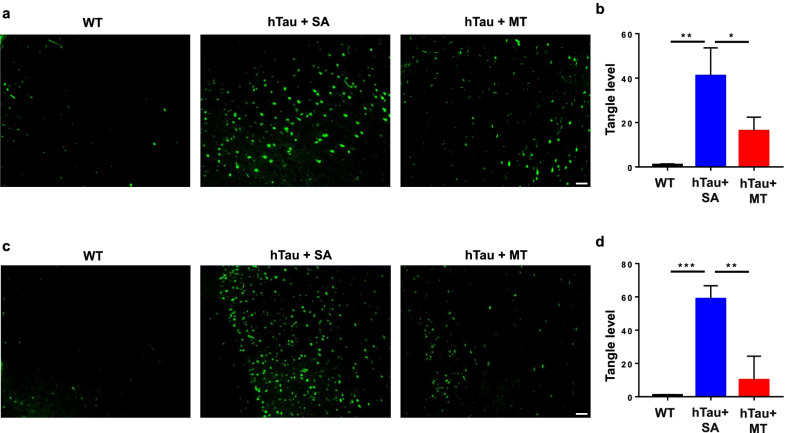
Melatonin decreases the formation of tangles. Brain sections from 10-month-old (**a, b**) and 13-month-old (**c, d**) WT (*n* = 8), hTau + SA (*n* = 5) and hTau + MT mice (*n* = 5) were stained with thioflavin-S to examine NFTs. Scale bars, 100 μm. Data are presented as the means ± standard errors (*P < 0.05, **P < 0.01, ***P < 0.001)

Considering that the accumulation of NFTs, which are composed of aggregated hyperphosphorylated tau, is a common hallmark of AD pathology, we next asked whether melatonin can decrease the formation of NFTs. The brains of hTau mice treated with saline exhibited a denser distribution of NFTs than the WT mice at 10 months of age, and the number of NFTs was further increased in 13-month-old hTau mice (Fig. 6). Treatment with melatonin decreased the number of NFTs by more than 50% at both 10 (Fig. 6a, b) and 13 (Fig. 6c, d) months of age.

**Fig. 7:**
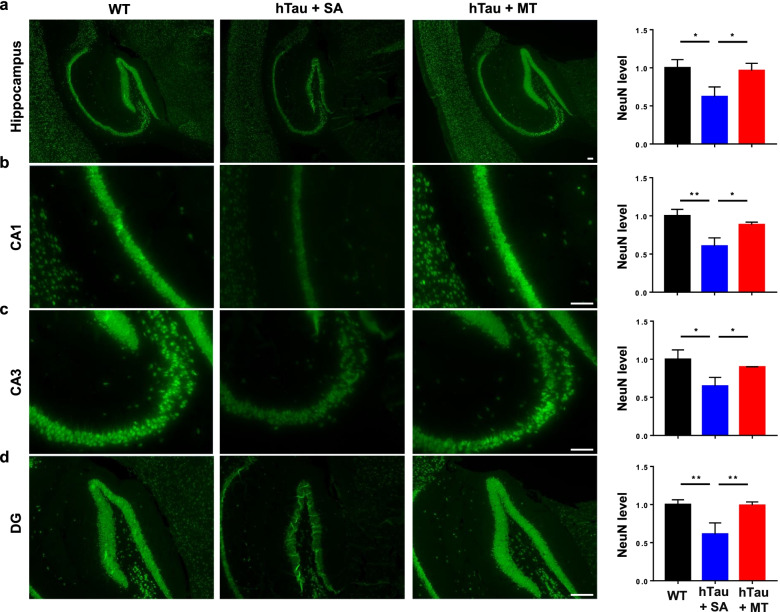
Melatonin decreases neuronal loss. The expression of NeuN was assessed to measure the number of neurons in the hippocampi of 13-month-old WT (*n* = 8), hTau + SA (*n* = 5) and hTau + MT mice (*n* = 5) (**a**). The CA1 (**b**), CA3 (**c**) and DG (**d**) regions of the hippocampus are shown. Scale bars, 100 μm. Data are presented as the means ± standard errors (*P < 0.05, **P < 0.01)

Tau hyperphosphorylation and aggregation have been reported to induce neuronal cell death [59, 60]. To investigate whether melatonin reduces neuronal cell death in hTau mice, we examined the number of neurons in hippocampal tissues by NeuN staining. There was no neuronal loss in 10-month-old hTau mice (data not shown), as previously reported [57]. At 13 months of age, the number of NeuN-positive cells in the hippocampi of hTau mice treated with saline was significantly reduced compared to the WT mice (Fig. 7a–d). However, the numbers of NeuN-positive neurons in the hippocampal CA1 (Fig. 7b), CA3 (Fig. 7c) and the dentate gyrus (DG) (Fig. 7d) were dramatically increased in the melatonin-treated hTau mice compared with the saline-treated hTau mice. These results clearly show that melatonin alleviates neuronal cell death in the hTau mice.

### Melatonin alters the miRNA expression profiles while attenuating tau pathology

**Fig. 8:**
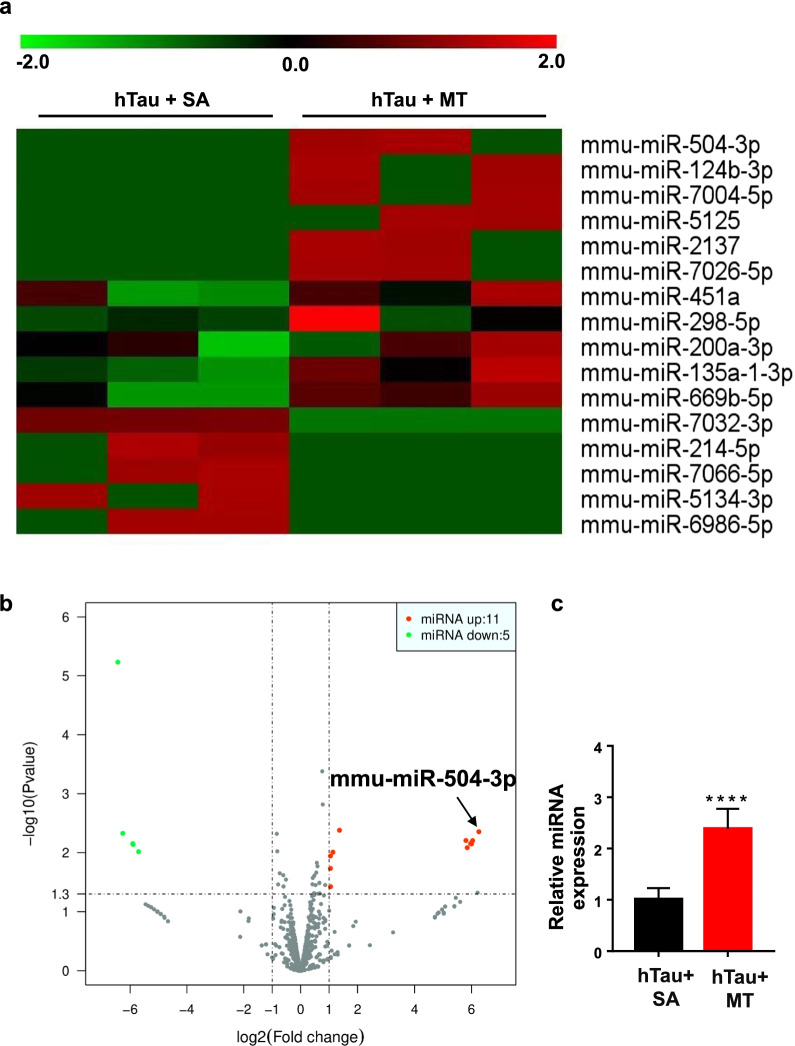
Melatonin alters the miRNA expression profile in hTau mice. MiRNA sequencing in the hippocampal tissues from hTau mice treated with saline or melatonin was performed using microarray (male, *n* = 3/group). The identified differentially expressed miRNAs between the two groups are displayed in the heatmap (**a**) and the volcano plot (**b**) (fold change > 2, *P* < 0.04). The mean fluorescence intensity was calculated as the average of three replicates. Eleven miRNAs were upregulated, while five miRNAs were downregulated. **c** The relative expression of miR-504-3p in the hTau mice treated with melatonin was confirmed by qRT-PCR. Data are presented as the means ± standard errors (****P < 0.0001 *vs.* the hTau + SA group)

We next examined the molecular mechanisms underlying the effects of melatonin in hTau mice. We performed high-throughput sequencing to compare miRNA expression in the brains of hTau mice treated with saline versus melatonin to study whether miRNAs are associated with the attenuation of tau pathology by melatonin. A total of 1978 miRNAs were analyzed in the microarray. The miRNAs with a fold change > 2 and *P*-value < 0.04 between the two groups were considered to be differentially expressed miRNAs. Sixteen differentially expressed miRNAs were identified, including 11 significantly overexpressed and 5 downregulated in the melatonin-treated hTau mice, as shown in the heatmap and the volcano plot (Fig. 8a, b). The volcano plot showed that mmu-miR-504-3p was the most dramatically differentially expressed miRNA, as it had the highest fold change and lowest *P* value. Therefore, miR-504-3p was selected for validation using qRT-PCR. Consistently, the qRT-PCR results showed that miR-504-3p was significantly overexpressed in the brains of melatonin-treated hTau mice compared with saline-treated hTau mice (Fig. 8c).

### Melatonin inhibits p39 via miR-504-3p

**Fig. 9:**
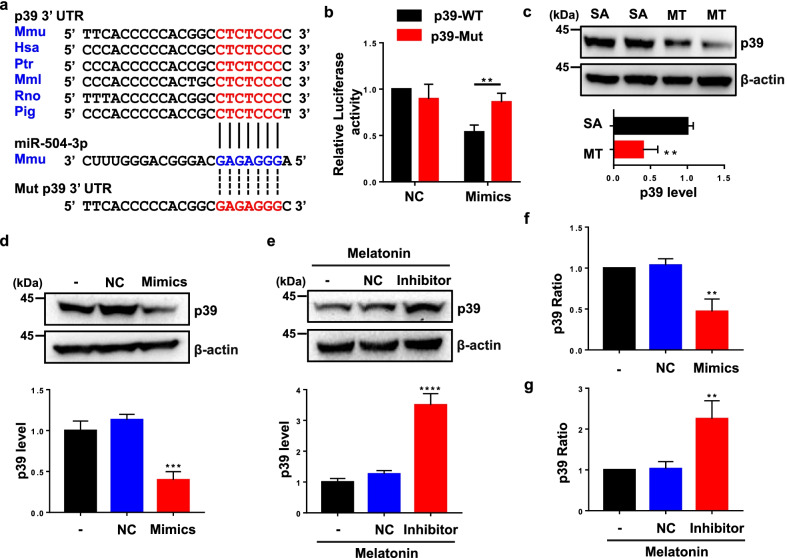
MiR-504-3p targets p39. **a** Sequences of the WT and mutant 3′UTR of p39. The 3′UTR of p39 harbors a miR-504-3p-binding site. **b** Results of the luciferase reporter assay using N2a cells cotransfected with a WT or mutant p39 3′UTR plasmid and miR-504-3p mimics or NC mimics. Data are presented as the means ± standard errors of three independent experiments (**P < 0.01 *vs.* the WT p39 group). **c** Hippocampal lysates from 10-month-old hTau + SA and hTau + MT mice were subjected to immunoblotting analysis with an anti-p39 or anti-β-actin antibody. **d, f** N2a cells were transduced with NC or miR-504-3p mimics. The protein (**d**) and mRNA (**f**) expression of p39 were measured by immunoblotting and qRT-PCR, respectively. β-actin was used as an internal control for immunoblotting, while 18S rRNA was used as an internal reference for qRT-PCR. Data are presented as the means ± standard errors of three independent experiments (**P < 0.01, ***P < 0.001 *vs.* the untreated group). **e, g** N2a cells were transduced with NC or a miR-504-3p inhibitor and then treated with melatonin. The protein (**e**) and mRNA (**g**) expression of p39 were measured by immunoblotting and qRT-PCR, respectively. Data are presented as the means ± standard errors of three independent experiments (**P < 0.01, ****P < 0.0001 *vs.* the group treated with melatonin alone)

Next, we performed bioinformatics analysis to predict the target genes of miR-504-3p. We found that miR-504-3p can target p39, which is an activator of CDK5 [14, 61]. As shown in Fig. 9a, the 3′UTR of p39 harbors a binding site for miR-504-3p. To confirm whether the 3′UTR of p39 is a functional target of miR-504-3p, we introduced a WT or mutant p39 3′UTR fragment into a luciferase reporter vector (pmirGLO) (Fig. 9a), which was then cotransfected with miR-504-3p mimics or NC mimics into N2a or 293T cells. We found that the miR-504-3p mimics significantly reduced the luciferase activity in the WT-3′UTR group, whereas mutation of the miR-504-3p-binding site abolished the reduction in luciferase activity, as determined by a dual-luciferase reporter gene assay (Fig. 9b and Additional file 1: Fig. S1). These results demonstrate that miR-504-3p specifically targets the 3′UTR of p39.

To investigate whether melatonin suppresses p39 expression by upregulating miR-504-3p expression, we examined the protein levels of p39 in brain lysates of hTau mice treated with saline or melatonin, in untreated cells transfected with miR-504-3p mimics, and in melatonin-treated cells transfected with a miR-504-3p inhibitor. The data showed that p39 expression was dramatically decreased in the melatonin-treated hTau mice compared with the saline-treated hTau mice (Fig. 9c) and in the miR-504-3p mimic group compared to the NC mimic group, while the melatonin-treated cells transfected with the miR-504-3p inhibitor had higher level of p39 compared with those transfected with the NC inhibitor (Fig. 9d, e). To further clarify whether miR-504-3p inhibits the expression of p39 by downregulating its mRNA expression, we investigated the mRNA levels of p39. Transfection of untreated cells with miR-504-3p mimics significantly reduced the mRNA expression of p39 compared to transfection of NC mimics, and the mRNA levels of p39 were increased in melatonin-treated cells transfected with the miR-504-3p inhibitor compared to cells transfected with the NC inhibitor (Fig. 9f, g). Thus, miR-504-3p suppresses p39 expression by regulating its mRNA level. Taken together, these results suggest that melatonin inhibits the expression of p39 by upregulating miR-504-3p expression.

### MiR-504-3p attenuates tau hyperphosphorylation by targeting p39

**Fig. 10:**
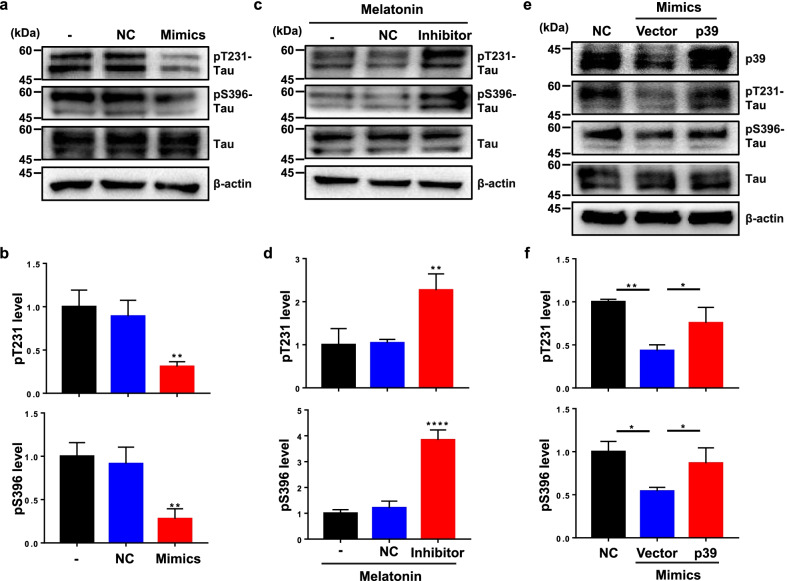
MiR-504-3p alleviates tau hyperphosphorylation by targeting p39. **a, b** N2a cells were transfected with NC or miR-504-3p mimics. The expression of pThr231 tau, pSer396 tau and total tau was examined. β-actin was used as an internal control. Data are presented as the means ± standard errors of three independent experiments (**P < 0.01 *vs.* the untreated group). **c, d** N2a cells were transfected with NC or a miR-504-3p inhibitor and then treated with melatonin. The expression of pThr231 tau, pSer396 tau and total tau was examined. β-actin was used as an internal control. The data are presented as the means ± standard errors of three independent experiments (**P < 0.01, ****P < 0.0001 *vs.* the group treated with melatonin alone). **e, f** N2a cells were cotransfected with NC or miR-504-3p mimics and a p39 plasmid (without a miR-504-3p-binding site) or vector. The protein levels of p39, pThr231 tau, pSer396 tau and total tau were measured using immunoblotting. Data are presented as the means ± standard errors of three independent experiments (*P < 0.05, **P < 0.01 *vs.* the group cotransfected with miR-504-3p mimics and vector)

Our results showed that melatonin decreases tau phosphorylation and NFTs and increases the expression of miR-504-3p, and miR-504-3p decreases the expression of p39, which is an activator of CDK5. Since CDK5 phosphorylates tau at multiple sites, including Thr231 and Ser396 [8, 13], we next asked whether miR-504-3p regulates tau phosphorylation. N2a cells were transfected with miR-504-3p mimics, and the phosphorylation levels of tau were examined with pThr231 and pSer396 tau antibodies. We found that the phosphorylated tau level, but not the total tau level, was dramatically lower in cells overexpressing miR-504-3p mimics than in cells in the untreated and NC mimic groups (Fig. 10a, b), indicating that miR-504-3p reduces tau phosphorylation at the CDK5-dependent phosphorylation sites related to AD. Moreover, the phosphorylation level of tau in the melatonin-treated cells transfected with the miR-504-3p inhibitor was significantly higher than that in the melatonin-treated cells transfected with NC (Fig. 10c, d). Next, we examined CDK5 activity by an in vitro kinase assay using its substrate APP because CDK5 phosphorylates APP at the Thr668 site [62]. While the miR-504-3p mimics decreased the phosphorylation of APP, melatonin with the miR-504-3p inhibitor increased APP phosphorylation (Additional file 1: Fig. S2). Neither of them affected CDK5 expression, suggesting that miR-504-3p regulates the CDK5 activity (Additional file 1: Fig. S2, input lanes). These data suggest that inhibition of miR-504-3p abrogates the effects of melatonin in alleviating tau hyperphosphorylation.

To investigate whether melatonin-mediated increase of miR-504-3p expression suppresses tau hyperphosphorylation by silencing p39, we rescued the expression of p39 in N2a cells overexpressing miR-504-3p by transfection of a plasmid expressing p39 without the 3′UTR. The data showed that cotransfection of miR-504-3p mimics and the p39 plasmid restored p39 expression (Fig. 10e, f). The levels of tau phosphorylated at Thr231 and Ser396 were increased in the p39 restoration group compared to the p39 silencing group (Fig. 10e, f). Therefore, restoring p39 expression in cells transfected with miR-504-3p mimics antagonizes the effects of miR-504-3p in attenuating tau hyperphosphorylation. These results indicate that the melatonin-induced up-regulation of miR-504-3p results in attenuation of tau hyperphosphorylation by silencing p39.

## Discussion

Although there is evidence for a critical role for melatonin in tau hyperphosphorylation and AD, whether or how melatonin regulates tau-related pathologies through miRNAs has not been well studied. Here, we demonstrated for the first time that melatonin alleviates tau hyperphosphorylation and tau-mediated neuronal loss in AD by elevating the expression of miR-504-3p in mice overexpressing WT human tau. Mechanistically, the increase in miR-504-3p expression induced by melatonin inhibits the CDK5-related tau phosphorylation by targeting p39, an activator of CDK5. The present study provides insight into the mechanism of melatonin-mediated attenuation of tau hyperphosphorylation in AD and may provide new ideas for the early diagnosis and treatment of AD.

We first showed that melatonin efficiently reduces tau phosphorylation, formation of NFTs and neuronal loss in hTau mice that express all six isoforms of human tau [57]. This finding provides more reliable evidence for the ability of melatonin to attenuate neurodegeneration, as the hTau mice show AD pathologies similar to those seen in humans. Aberrant tau phosphorylation is one of the earliest events in AD [63–65]. Hyperphosphorylation of tau results in tau aggregation. The sarkosyl-insoluble fraction, which is enriched in abnormally phosphorylated tau and fibrils, is important for abnormal tau aggregation [5–8]. Our data showed that the tau phosphorylation levels were decreased to a much greater extent in the sarkosyl-insoluble fraction than in the sarkosyl-soluble fraction in the melatonin-treated hTau mice. These results suggest that melatonin mainly reduces tau aggregation. In addition, both the total and the phosphorylated tau levels were dramatically decreased in the sarkosyl-insoluble fraction in the melatonin-treated group compared with the saline-treated group, whereas only phosphorylated tau levels were reduced in the sarkosyl-soluble fraction in the melatonin-treated mice compared with the saline-treated mice. It has been recently reported that melatonin directly interacts with the repeated domain of tau that is critical for the association with microtubules and tau aggregation. The direct binding of melatonin and tau inhibits tau aggregation [66]. These data indicate that melatonin may suppress the pathological transformation of tau from the normal soluble form to the abnormal filamentous insoluble form through multiple mechanisms, including direct binding to tau or regulation of its downstream targets. The data indicate that the phosphorylation level of tau was not significantly different between 10- and 13-month-old hTau mice. There were slightly more NFTs in 13-month-old hTau mice than in 10-month-old hTau mice. These results are consistent with the finding that hyperphosphorylated tau starts to accumulate by six months of age and that the hyperphosphorylated tau accumulation increases further by 13 months in hTau mice [57]. However, neuronal cell death was dramatically induced in hTau mice compared with WT mice at 13 months of age but not at 10 months of age. A previous study has reported neuronal decrease at 14 months of age compared to 10 months in hTau mice [67]. Therefore, it can be speculated that neuronal cell death does not occur in hTau mice at 10 months of age. In this study, we administered melatonin to 5- and 8-month-old hTau mice for 5 months and found that early and late treatment with melatonin efficiently decreased the number of tau-containing NFTs and neuronal loss, indicating that melatonin can reduce tau-related pathology, even when administered at a late time point. More studies are required to determine whether melatonin has similar effects in older hTau mice and rescues cognitive impairment in this animal model.

We discovered that melatonin significantly upregulated the expression of 11 miRNAs and downregulated the expression of 5 miRNAs in the hTau mouse brain. Among the 11 increased miRNAs, miR-504-3p displayed the highest upregulation in response to melatonin administration. Previous research has shown that miR-504-3p expression is decreased in the brains of human patients with AD compared with those of normal subjects [68]. It has also been shown that the levels of melatonin are decreased in the AD brains [27, 30] and that CSF melatonin levels are negatively correlated with the Braak stage for tangles [27]. Whether miR-504-3p expression is decreased to a greater extent in severe AD than in mild AD and whether miR-504-3p is implicated in the pathogenesis of AD are worth exploring. Moreover, it has been shown that melatonin levels are significantly reduced in aged individuals with early AD-related neuropathological changes, which suggests that the decreased melatonin levels in the CSF might be a biomarker for the early stage of AD [27]. Thus, we propose that a reduction in miR-504-3p expression might also be an early event in the development of AD.

CDK5 has been proposed as a major protein kinase that regulates tau hyperphosphorylation in AD and is a particularly interesting protein kinase due to its critical role in the pathogenesis of AD and in manipulating other tau kinases, including glycogen synthase kinase 3β [69, 70]. CDK5 knockdown reduces tau phosphorylation and the number of NFTs in AD [71, 72]. CDK5, a member of the proline-directed serine/threonine cyclin-dependent kinase family, is activated through interaction with its activators p35 and p39 or their cleavage products (p25 and p29, respectively) [15, 61, 69, 73]. CDK5 association with p39 is thought to have a higher ability to phosphorylate tau than p35 [14]. This study showed that melatonin decreases the expression of p39 via upregulation of miR-504-3p expression, leading to a reduction in tau phosphorylation at sites related to CDK5. It has been demonstrated that p25 and p29 are more stable than p35 and p39, respectively, allowing prolonged activation of CDK5 [74, 75]. Melatonin reduces the expression and activity of CDK5 by preventing the cleavage of p35 to p25, which results in the formation of a stable and active complex between CDK5 and p25 [76]. Thus, more studies are required to explore whether melatonin decreases the cleavage of p39 to p29.

Our results strongly indicate that melatonin may protect against the development of AD and alleviate tau-related pathologies in a preventive as well as in a curative manner in an animal model. Melatonin is able to cross the blood–brain barrier and has been used to improve the sleep quality of AD patients with low toxicity and high bioavailability [22, 77]. The combined application of a controlled dose of melatonin and miR-504-3p mimics or p39/CDK5 inhibitors may provide novel therapeutic options for the treatment of AD. Future studies are expected to investigate the effects of miR-504-3p mimics or miR-504-3p inhibitors with melatonin in hTau transgenic mice or AD-like animal models and the translational value of miR-504-3p as a biomarker and therapeutic target for AD.

## Conclusions

**Fig. 11:**
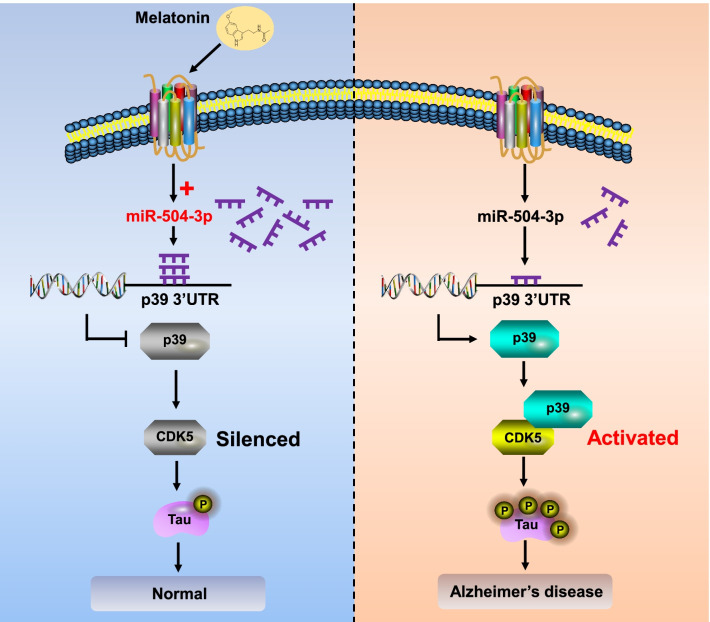
Melatonin attenuates tau hyperphosphorylation via the miR-504-3p/CDK5/p39 axis in AD. Under normal conditions (left), melatonin increases the expression of miR-504-3p, which targets p39 and inhibits the activity of CDK5, resulting in physiological phosphorylation of tau. In AD (right), loss of melatonin leads to downregulation of miR-504-3p expression and overexpression of p39, which promotes the interaction between p39 and CDK5. Furthermore, activation of CDK5 ultimately promotes tau hyperphosphorylation and tau-related pathology in the AD brain

In summary, we propose a novel mechanism by which melatonin mediates upregulation of miR-504-3p, leading to transcriptional downregulation of p39 in hTau mice, modulating the phosphorylation of tau protein at CDK5-dependent phosphorylation sites in AD (Fig. 11). Our study thus demonstrates a critical role for melatonin in regulating miRNA expression and tau-related pathologies and may offer novel therapeutic strategies for AD.

## Supplementary Information

**Additional file 1:** **Table S1.** Primary antibodies used in the present study. **Fig. S1.** MiR-504-3p binds to the p39 3′UTR. **Fig. S2.** Melatonin decreases CDK5 activity through the miR-504-3p/p39 axis.

**Additional file 2:** MicroRNA expression profile dataset.

## Data Availability

The datasets generated and/or analyzed in the present study are available from the corresponding author upon reasonable request.

## Abbreviations

Aβ: Beta-amyloid
AD: Alzheimer's disease
CDK5: Cyclin-dependent kinase 5
CSF: Cerebrospinal fluid
DG: Dentate gyrus
MARK: Microtubule affinity-regulating kinase
miRNA: MicroRNA
NFT: Neurofibrillary tangle
WT: Wild-type
