# Supplementary material for: Melatonin ameliorates tau-related pathology via the miR-504-3p and CDK5 axis in Alzheimer’s disease
Source: Transl Neurodegener. 2022 May 9;11:27. doi: 10.1186/s40035-022-00302-4 (PMC9082841; doi:10.1186/s40035-022-00302-4)
Supplement: Supplementary file 1 — Additional file 1: Table S1. Primary antibodies used in the present study. Fig. S1. MiR-504-3p binds to the p39 3′UTR. Fig. S2. Melatonin decreases CDK5 activity through the miR-504-3p/p39 axis. [file 40035_2022_302_MOESM1_ESM.pdf]

Additional Files 1

Chen et al.

Melatonin ameliorates tau-related pathology via the miR-504-3p and CDK5 axis in Alzheimer's disease

Table S1. Primary antibodies used in the present study.

Antibodies	Dilutions	Source	Identifier
Mouse anti-β-actin	1:40000 (WB)	MilliporeSigma	A5441
Rabbit anti-p39	1:8000 (WB)	Abcam	ab124896
	1:1000 (WB)	Cell Signaling Technology	3275S
	1:1000 (WB)	Thermo Fisher Scientific	PA5-103024
Rabbit anti-CDK5	1:1000 (WB)	Cell Signaling Technology	2506S
Rabbit anti-pT668-APP	1:1000 (WB)	Cell Signaling Technology	3823S
Mouse anti-Tau	1:1000 (WB)	Cell Signaling Technology	4019S
Mouse anti-pS202/T205-Tau	1:150 (IF)	Thermo Fisher Scientific	ENMN1020
Rabbit anti-pT231-Tau	1:5000 (WB)<br>1:1000 (IF)	Abcam	ab151559
Rabbit anti-pS262-Tau	1:1000 (WB)	Thermo Fisher Scientific	44750G
Rabbit anti-pS396-Tau	1:3000 (WB)	Anaspec	AS-54977

WB, Western blotting; IF, Immunofluorescent.

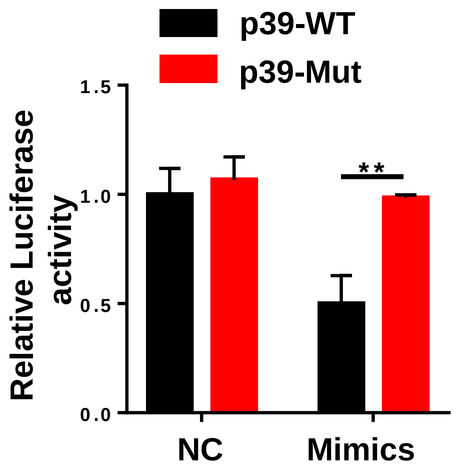

Fig. S1 MiR-504-3p binds to the p39 3’UTR.

Results of luciferase reporter assays using 293T cells cotransfected with a WT or mutant p39 3’UTR plasmid and miR-504-3p mimics or NC mimics. The data are presented as the means ± standard errors of three independent experiments (***P* < 0.01 *vs.* the WT p39 group).

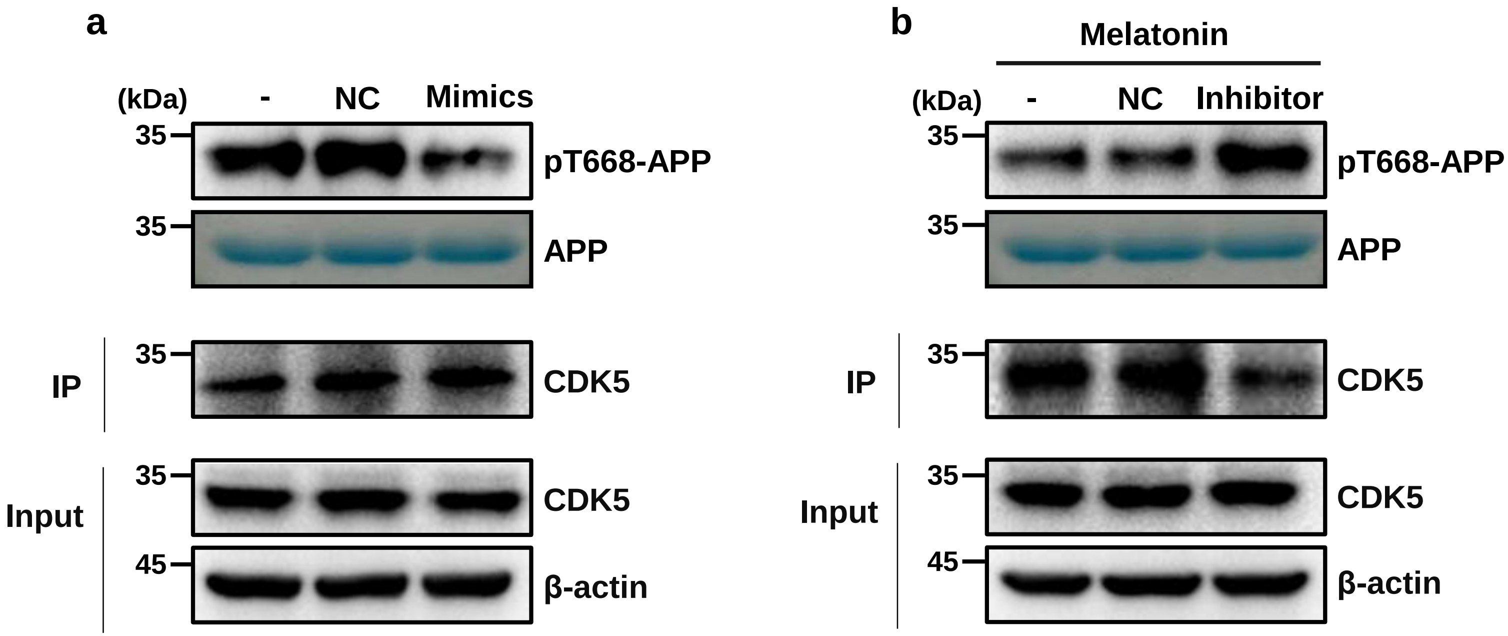

Fig. S2 Melatonin decreases CDK5 activity through the miR-504-3p/p39 axis.

N2a cells were transduced with NC or miR-504-3p mimics or with NC or miR-504-3p inhibitors and then treated with melatonin. The protein samples were subjected to immunoblotting analysis with an anti-CDK5 or anti-β-actin antibody. N2a cell lysates were subjected to immunoprecipitation with an anti-CDK5 antibody, followed by immunoblotting analysis with an anti-pThr668 APP or an anti-CDK5 antibody. The total level of GST-C-terminal APP protein is shown by Coomassie brilliant blue staining.
